# How Does Fingolimod (Gilenya^®^) Fit in the Treatment Algorithm for Highly Active Relapsing-Remitting Multiple Sclerosis?

**DOI:** 10.3389/fneur.2013.00010

**Published:** 2013-05-01

**Authors:** Franz Fazekas, Ovidiu Bajenaru, Thomas Berger, Tanja Hojs Fabjan, Alenka Horvat Ledinek, Gábor Jakab, Samuel Komoly, Tetiana Kobys, Jörg Kraus, Egon Kurča, Theodoros Kyriakides, L'ubomír Lisý, Ivan Milanov, Tetyana Nehrych, Sergii Moskovko, Panayiotis Panayiotou, Saša Šega Jazbec, Larysa Sokolova, Radomír Taláb, Latchezar Traykov, Peter Turčáni, Karl Vass, Norbert Vella, Nataliya Voloshyná, Eva Havrdová

**Affiliations:** ^1^Department of Neurology, Medical University of GrazGraz, Austria; ^2^Department of Neurology, Neurosurgery and Psychiatry, University of Medicine and Pharmacy “Carol Davila,”Bucharest, Romania; ^3^Department of Neurology, Medical University of InnsbruckInnsbruck, Austria; ^4^Department of Neurology, University Medical Center MariborMaribor, Slovenia; ^5^Department of Neurology, University Medical Center LjubljanaLjubljana, Slovenia; ^6^Department of Neurology, Uzsoki HospitalBudapest, Hungary; ^7^Department of Neurology, University of PécsPécs, Hungary; ^8^MS HospitalKyiv, Ukraine; ^9^Department of Neurology, Salzburger Landeskliniken, Paracelsus Medical UniversitySalzburg, Austria; ^10^Department of Neurology, University HospitalMartin, Slovakia; ^11^Neuropathology Lab, Clinic A, Cyprus School of Molecular Medicine, Cyprus Institute of Neurology and GeneticsNicosia, Cyprus; ^12^Department of Neurology, University Hospital of Bratislava – Ruzinov HospitalBratislava, Slovakia; ^13^Neurologic Clinic, University Hospital “Saint Naum,”Sofia, Bulgaria; ^14^Neurology Department, National Medical UniversityLviv, Ukraine; ^15^Neurology Department, National Medical UniversityVinnytsya, Ukraine; ^16^Aretaieio Private HospitalNicosia, Cyprus; ^17^Department of Neurology, University Medical Center LjubljanaLjubljana, Slovenia; ^18^Department of Neurology, National Medical UniversityKyiv, Ukraine; ^19^Department of Neurology, Hradec Kralove HospitalHradec Kralove, Czech Republic; ^20^Neurology Clinic, University Hospital AlexandrovskaSofia, Bulgaria; ^21^Department of Neurology, Comenius UniversityBratislava, Slovakia; ^22^Department of Neurology, Medical University of ViennaVienna, Austria; ^23^Department of Neurology, Mater Dei Hospital Tal-QroqqMsida, Malta; ^24^Department of Neuroinfection, Institute of Neurology, Psychiatry and Narcology, AMS UkraineKharkiv, Ukraine; ^25^Department of Neurology, First Medical Faculty, Charles University in PraguePrague, Czech Republic

**Keywords:** multiple sclerosis, fingolimod, treatment, algorithm, expert opinion

## Abstract

Multiple sclerosis (MS) is a neurological disorder characterized by inflammatory demyelination and neurodegeneration in the central nervous system. Until recently, disease-modifying treatment was based on agents requiring parenteral delivery, thus limiting long-term compliance. Basic treatments such as beta-interferon provide only moderate efficacy, and although therapies for second-line treatment and highly active MS are more effective, they are associated with potentially severe side effects. Fingolimod (Gilenya^®^) is the first oral treatment of MS and has recently been approved as single disease-modifying therapy in highly active relapsing-remitting multiple sclerosis (RRMS) for adult patients with high disease activity despite basic treatment (beta-interferon) and for treatment-naïve patients with rapidly evolving severe RRMS. At a scientific meeting that took place in Vienna on November 18th, 2011, experts from ten Central and Eastern European countries discussed the clinical benefits and potential risks of fingolimod for MS, suggested how the new therapy fits within the current treatment algorithm and provided expert opinion for the selection and management of patients.

## Introduction

Multiple sclerosis (MS) is a chronic, immune-mediated disease of the central nervous system (CNS) in which autoreactive CD4+ and CD8+ T lymphocytes, B lymphocytes, macrophages, antibodies, and cytokines attack the myelin sheaths and damage the axons. MS appears in distinct disease courses, the most common of which shows a waxing and waning of neurological symptoms and signs, and is thus termed relapsing-remitting MS.

The standard disease-modifying drugs (DMDs) such as interferon-beta and glatiramer acetate provide moderate efficacy and a low to moderate frequency of side effects. Second-line therapies for highly active MS such as the humanized antibody natalizumab and the cytostatic agent mitoxantrone are more effective, but associated with potentially severe side effects [e.g., progressive multifocal leukoencephalopathy (PML), cardiotoxicity, acute leukemia]. All these currently available treatments for MS require regular and frequent parenteral administration and may therefore interfere with long-term compliance. Fingolimod (Gilenya^®^) is the first oral treatment of MS with a completely new mode of action targeting not only inflammation but potentially also neurodegeneration. It has recently been approved as single disease-modifying therapy in highly active relapsing-remitting MS (RRMS) for adult patients with high disease activity despite basic treatment (beta-interferon) and for treatment-naïve patients with rapidly evolving severe RRMS. The approval was based on the largest phase III clinical trial program in MS at the time of submission.

The increasing information on the pathophysiology of both initial and later stages of the disease and the fact that tissue damage is irreversible mean that an early start to an effective therapy is the only way to obtain good results in the treatment of MS. It has to be taken into account that only 30% of MS patients are full responders to first-line treatment and also that the risk/benefit ratio changes with the severity of the disease. Furthermore, at least 30% of patients receiving immunomodulatory treatment have at least one relapse per year and disease activity as shown by MRI indicates that a switch to another medication could be helpful (Mäurer et al., 2011). Often, the exposure to ineffective first-line drugs is unacceptably long. Therefore a decision not to escalate early for economic reasons may be wrong and short-sighted because disability is incrementally expensive. For a reasonable therapy algorithm clinical evidence as well as considerations about the individual benefit/risk-balance, the patient’s wishes, and last but not least also economic aspects have to be taken into account.

**Figure 1:**
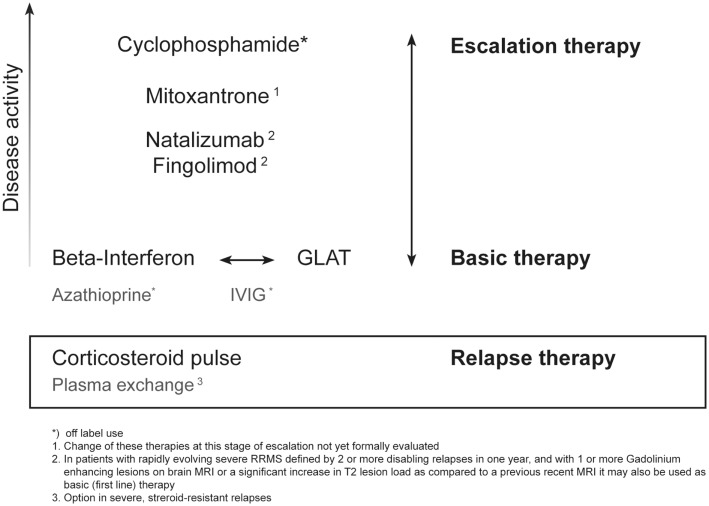
**Current options of escalating immunotherapy for RRMS**.

At a scientific meeting that took place in Vienna on November 18th, 2011, experts from ten Central and Eastern European countries discussed the clinical benefits and potential risks of fingolimod for MS, suggested how the new therapy fits within the current treatment algorithm (Figure 1 modified from Fazekas, 2012) and gave recommendations for the selection and management of patients. In many instances these reflect meeting participants’ expert opinions due to the lack of scientific evidence in several issues at this stage.

## Indication

**Figure 2:**
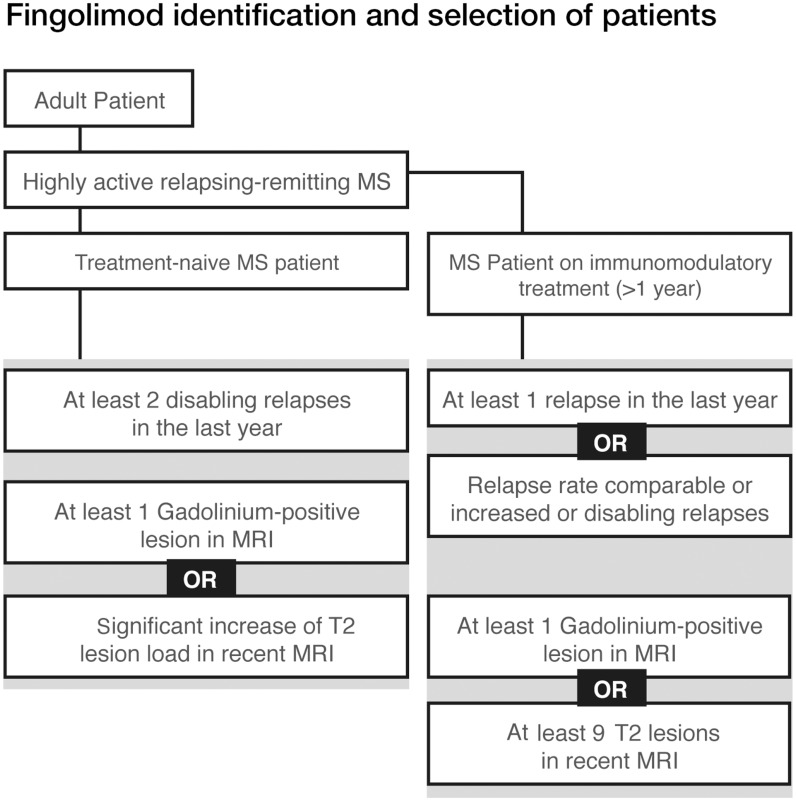
**Fingolimod identification and selection of patients**.

Whereas fingolimod is approved as first-line medication in the USA, in Switzerland, and in Australia, Europe has a more conservative approach. Here fingolimod is indicated and labeled as single disease-modifying monotherapy in highly active RRMS for the following adult patient groups (EU wide marketing authorization for Gilenya^®^ granted on 17 March 2011):

- Patients with high disease activity despite treatment with a beta-interferon. These patients may be defined as those who have failed to respond to a full adequate course (normally at least 1 year of treatment) of beta-interferon. Patients should have had at least one relapse in the previous year while on therapy, and have at least nine T2 hyperintensive lesions on cranial MRI, or at least one Gadolinium-enhancing lesion. A “non-responder” could also be defined as a patient with an unchanged or increased relapse rate or ongoing severe relapses, as compared to the previous year (Figure 2).
- Patients with rapidly evolving severe RRMS without prior treatment, defined by two or more disabling relapses in 1 year, and with one or more Gadolinium-enhancing lesions on brain MRI or a significant increase in T2 lesion load as compared to a recent MRI.
- From a clinical point of view treatment failure is defined as continuing disease activity (in the form of relapses supported by new or active MRI lesions) and progression in disability.
- According to expert opinion these definitions would similarly apply to prior treatment with glatiramer acetate.

In clinical practice unbearable side effects and low compliance may be considered as another kind of treatment failure.

## Mode of Action

Fingolimod is the first-in-class sphingosine 1-phosphate receptor (S1PR) modulator that binds with different affinities to four of the five known receptors (S1P_1_, S1P_3_, S1P_4_, and S1P_5_). S1P has important functions in the immune system, CNS and cardiovascular system, and S1P signaling plays a key role in neuroinflammatory processes (Spiegel and Milstien, 2003; Brinkmann, 2007, 2009; Rivera et al., 2008; Aktas et al., 2010).

The binding of fingolimod-P to the receptor is followed by the internalization and degradation of the receptor-fingolimod complex resulting in a secondary loss of surface receptors. As a result an initial agonist activity turns into a functionally antagonistic pharmacological effect during long-term administration (Chun and Brinkmann, 2011).

Fingolimod’s main effect on the immune system is the down-modulation of S1P_1_ receptors in lymphocytes, which leads to a reversible retention of circulating lymphocytes in lymph nodes, reducing their recirculation to the CNS. Fingolimod selectively inhibits naïve and central memory T-cell egress from lymph nodes but spares effector memory T-cells (*p* < 0.005 vs. untreated patients; Mehling et al., 2008) and retains their functional capacity (Mehling et al., 2008, 2010) and therefore preserves key immune functions (defense against infection; Pham et al., 2008; Brinkmann, 2009; Chun and Hartung, 2010). It causes a redistribution, rather than depletion, reducing the infiltration of autoreactive lymphocytes into the CNS where they would be involved in inflammation and nervous tissue damage.

The different receptor binding explains why fingolimod has different pharmacodynamic properties from the classical immunosuppressants and is, thus, defined as a selective immunosuppressant (Table 1). It does not appear to inhibit the activation, proliferation, or memory formation of T-cells, nor to interfere with antibody formation by B cells, and cytokine synthesis by T-cells.

**Table 1:** **Immunomodulatory properties of fingolimod**.

The available data support the notion that fingolimod at its approved dose may not act as a potent immunosuppressant
Neuroprotective CNS effects unrelated to immunosuppression may be present
Suboptimal prevention of graft rejection was achieved in renal transplantation studies in combination with cyclosporine, despite being at 10× the approved MS dose
Immunological constituents are maintained (cellular and humoral), with reversible effects on cell location of some (but not all) lymphocyte subsets – without inhibition of proliferation, differentiation, and cytotoxicity
Immunological surveillance is maintained through relatively unaffected effector memory T-cells
Clinically, the overall incidence of infections as well as of serious and severe infections is not substantially increased

*Modified from Chun and Brinkmann (2011)*.

Based on its lipophilic nature fingolimod crosses the blood-brain barrier and the oral formulation can result in biologically active concentrations in the CNS (Foster et al., 2007).

There is a growing body of preclinical evidence to indicate that the drug also down-modulates S1P_1_ in CNS cells, e.g., astrocytes to reduce astrogliosis, a phenomenon associated with neurodegeneration in MS. This may help restore gap-junctional communication of astrocytes with neurons and cells of the blood-brain barrier. Additional effects may result from (down-) modulation of S1P_3_ in astrocytes and of S1P_1_ and S1P_5_ in oligodendrocytes. Thus, fingolimod may target the disease process of MS not only by reducing inflammation, but also by direct protective actions within the lesions.

## Clinical Data: Fingolimod in RRMS

### Efficacy

Two recent multinational phase III studies in RRMS, the TRANSFORMS (Cohen et al., 2010a), and FREEDOMS (Kappos et al., 2010) studies, compared fingolimod with interferon-beta-1a (IFN beta-1a), or placebo, respectively, with both demonstrating superior efficacy in terms of clinical and MRI outcomes.

**Figure 3:**
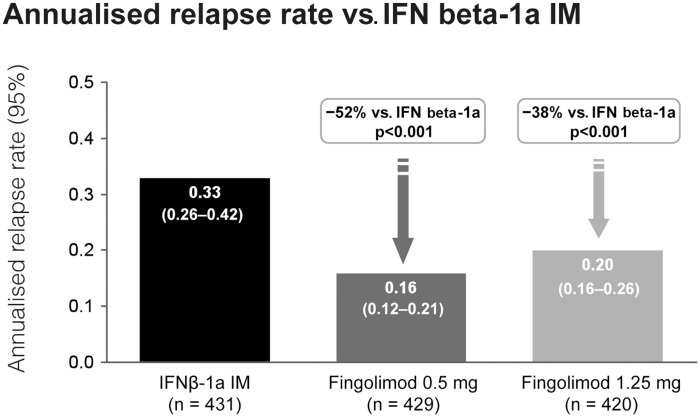
**Adjusted annualized relapse rate in the TRANSFORMS study comparing the efficacy of fingolimod with interferon-beta-1a i.m**.

**Figure 4:**
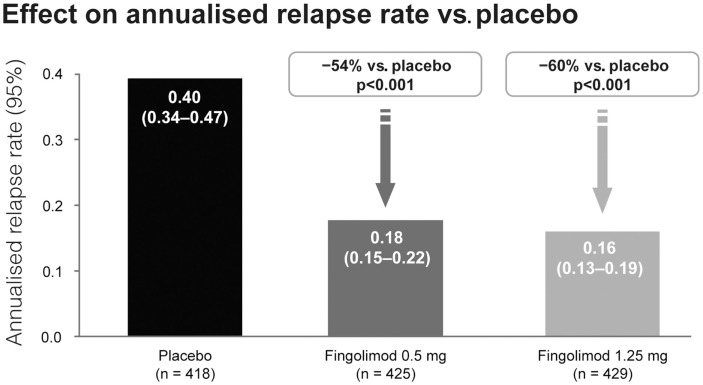
**Adjusted annualized relapse rate in the FREEDOMS study comparing the efficacy of fingolimod with placebo**.

Fingolimod treatment significantly reduced annualized relapse rate (ARR) vs. control in both studies (*p* < 0.001). In the TRANSFORMS study (*n* = 1292 patients) the relative reduction in ARR was 52% vs. IFN beta-1a at 1 year (Figure 3). About 83% of fingolimod-treated patients remained relapse-free, compared with 69% receiving IFN beta-1a (*p* < 0.001). In the FREEDOMS study (*n* = 1272) the relative reduction in ARR was 54% compared with placebo (Figure 4). After 2 years of therapy, 70% of the patients in the fingolimod group remained free from relapses (vs. 46% in the placebo group). Fingolimod 0.5 mg reduced the risk of disability progression confirmed after 3 and 6 months by 30% (*p* = 0.02) and 37% (*p* = 0.01), respectively, compared with placebo over 2 years. Patients who received IFN beta-1a in the TRANSFORMS study (months 0–12) had a significant reduction in ARR within 1 year of switching to fingolimod (months 13–24; −30%, *p* < 0.05; Cohen et al., 2010b). However, patients receiving fingolimod for 2 years continuously had an even greater reduction in ARR than patients receiving IFN beta-1a for 1 year before switching to fingolimod for the second year [ARR (95% CI) 0.33 (0.27–0.39) vs. 0.18 (0.14–0.22); relative risk reduction: −46%; *p* < 0.001].

During the FREEDOMS study EDSS scores remained stable, compared with baseline, in patients receiving fingolimod treatment (mean EDSS score ± SD, 0.0 ± 0.88) and worsened in the placebo group (0.13 ± 0.94; *p* = 0.002). Similarly, MS Functional Composite (MSFC) *z*-scores improved slightly in patients receiving fingolimod (0.03 ± 0.39), compared with baseline, and worsened in the placebo group (−0.06 ± 0.57; *p* = 0.01).

Subgroup analyses showed that fingolimod treatment had consistent effects, regardless of whether patients were treatment-naïve or had previously received disease-modifying therapy, and irrespective of gender, pre baseline disease activity, or disease history (Devonshire et al., 2012). Recent *post hoc* analyses from FREEDOMS and TRANSFORMS demonstrated that fingolimod is an effective treatment in treatment-naïve patients with severe disease (rapidly evolving severe RRMS defined as ≥2 relapses in the previous year and ≥1 Gd+ lesion at baseline). The ARR reduction was 67% at 2 years compared with placebo (*p* < 0.001) and 25% at 1 year compared with IFNβ-1a (*p* = 0.614; Havrdová et al., 2011; Devonshire et al., 2012).

### Effect on MRI measures

Fingolimod therapy demonstrated a highly significant reduction in inflammatory disease activity (T1 Gd-enhancement, new, or enlarging T2-lesions), burden of disease (volume of T2/PD hyperintensive lesions), and tissue loss and destruction [volume of T1 hypointense lesions (black holes)].

Fingolimod 0.5 mg significantly (*p* < 0.001) reduced the number of MRI inflammatory lesions vs. placebo (Kappos et al., 2010). After 2 years the relative reduction was 74% for new or enlarged T2-lesions (2.5 vs. 9.8) and 82% for Gd-enhancing T1 lesions (0.2 vs. 1.1). Patients treated with fingolimod 0.5 mg also had significantly fewer new or enlarged hyperintense lesions on T2 weighted images (1.7 vs. 2.6 for IFN beta-1a, RR = 35%) and fewer Gd-enhancing T1 lesions (0.23 vs. 0.51, RR = 55%) at 12 months compared to the IFN beta-1a group (Cohen et al., 2010a).

Patients who switched to fingolimod after receiving IFN beta-1a had further significant reductions in inflammatory lesion activity (Cohen et al., 2010b). The relative risk reduction was 66% for new or enlarged T2-lesions (2.1 for IFN beta-1a vs. 0.7 for fingolimod 0.5 mg; *p* < 0.001 for year 1 vs. year 2) and as much as 80% for Gd-enhancing T1 lesions (0.5 vs. 0.1; *p* < 0.002). Long-term phase II-data showed that these effects were sustained and did not diminish with long-term treatment (Antel et al., 2012).

Fingolimod treatment also resulted in a significant decrease in total lesion volumes with a relative reduction of 83% for T1 hypointense “black hole” lesion volume compared with placebo after 2 years (8.8 vs. 50.7%; *p* = 0.012). The relative reduction of T2 lesion volume (burden of disease) was 69% (10.6 vs. 33.8%; Cohen et al., 2010a; Radue et al., 2010).

**Figure 5:**
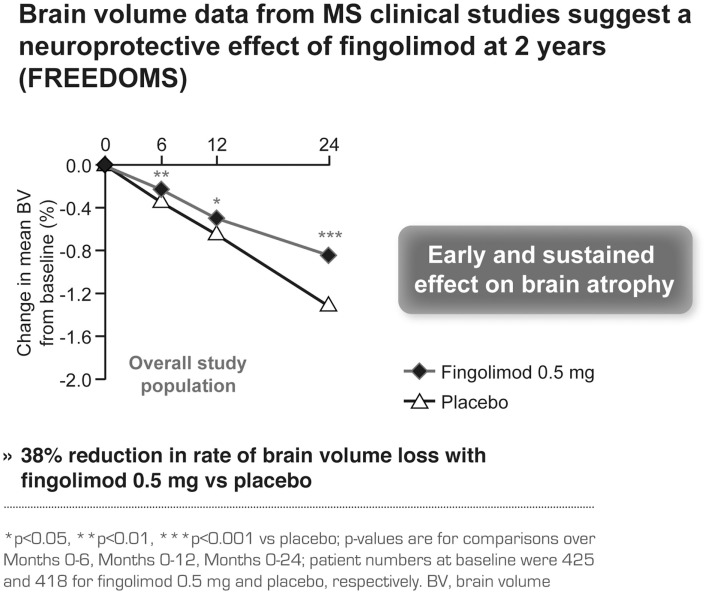
**Brain volume data in the FREEDOMS study comparing the efficacy of fingolimod with placebo**.

Brain volume data from MS clinical studies support the efficacy of fingolimod in preventing brain damage (Brinkmann, 2007). This effect was seen as early as Month 6 and was sustained until study end, independent of the inflammation status, resulting in a 38% reduction in the rate of brain volume loss with fingolimod 0.5 mg compared with placebo (*p* < 0.001; Figure 5; Radue et al., 2010). The brain volume data in FREEDOMS I were confirmed in FREEDOMS II (Kappos et al., 2012). Moreover, fingolimod led to a stronger reduction in the rate of brain atrophy over 1 year when compared to IFN beta-1a, irrespective of prior disease activity (Barkhof et al., 2011; Devonshire et al., 2012).

### Tolerability

**Figure 6:**
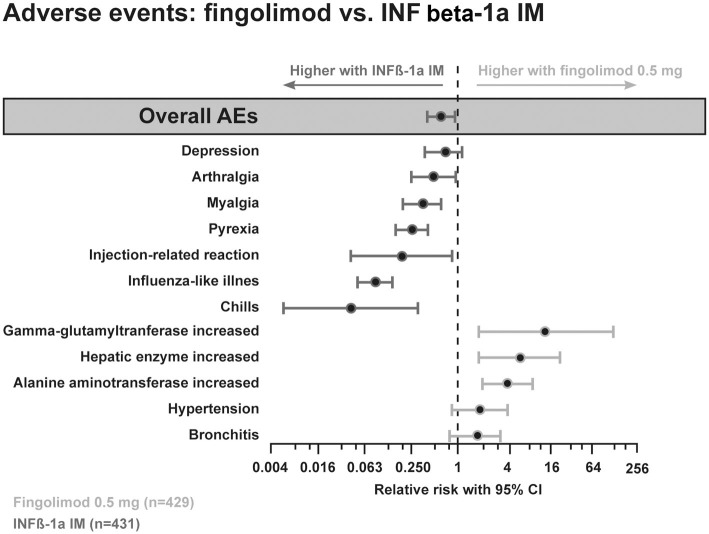
**Adverse event profile comparing the efficacy of fingolimod with interferon beta-1a i.m**.

The phase III studies reported similar fingolimod-related adverse events (AEs). These included transient, dose-dependent, generally asymptomatic cardiac events with the first dose, mild blood pressure increases, rare macular edema and asymptomatic, reversible elevation of liver enzymes (Figure 6). All observed side effects are well explained by the S1P-receptor binding properties of fingolimod.

**Figure 7:**
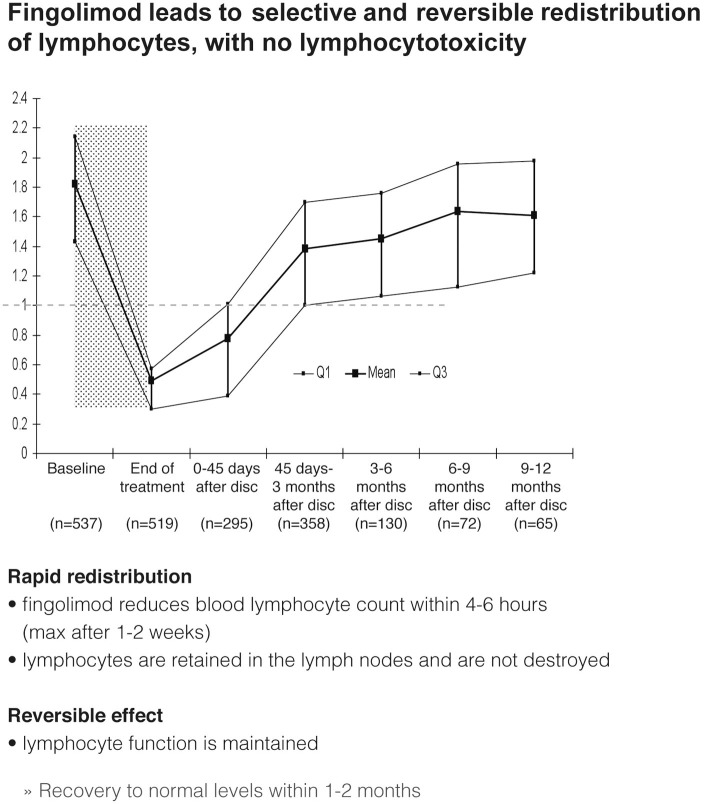
**Effects of fingolimod on lymphocyte counts and distribution after discontinuation (disc)**.

During treatment with fingolimod lymphocyte counts dropped rapidly within 4–6 h, approaching steady state levels in 2–4 weeks, and remained stable on continued treatment (Figure 7). Fingolimod leads to selective and reversible redistribution of lymphocytes without causing any lymphocytotoxicity. Lymphocytes are retained in the lymph nodes and are not destroyed (Francis et al., 2011). The lymphocyte function is maintained. A recovery to normal levels usually takes place within 1–3 months. Despite the reduction in lymphocyte counts, fingolimod-treated patients with MS were shown not to have more infections apart from a somewhat higher number of respiratory tract infections (Kappos et al., 2012) and were able to mount antigen-specific immune responses in vaccination studies (Kappos et al., 2011; Mehling et al., 2011).

## Managing Fingolimod in Clinical Practice

Fingolimod has been available in the EU since March 2011 and can be easily transferred from the extensive MS clinical study program to clinical practice. Four steps are considered essential for treating patients with fingolimod. The first step and probably the most important issue is the identification and selection of patients.

### Identification and selection of patients

#### Non-responder to IFN beta or glatiramer acetate (GA)

**Figure 8:**
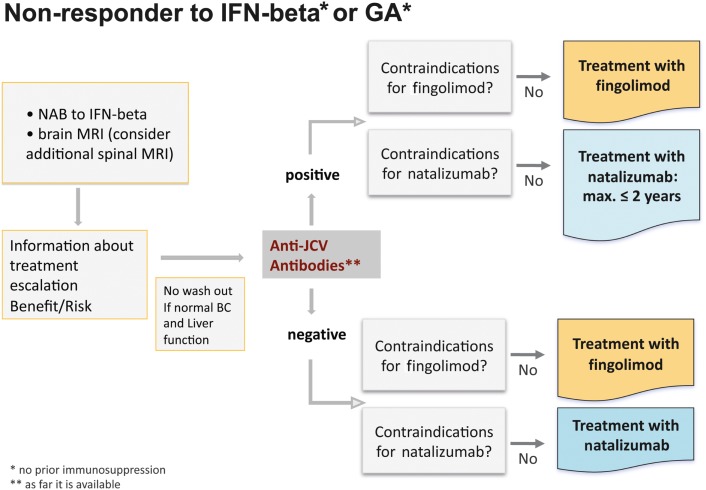
**Treatment algorithm for non-responders to interferon-beta or glatiramer acetate**.

Switching to an escalation therapy with either fingolimod or natalizumab is indicated for patients with high disease activity despite treatment with IFN beta or GA. Non-responders should have at least one relapse in the previous year while on therapy, and have at least 9 T2 hyperintense lesions in cranial MRT or at least one Gd-enhancing lesions (Figure 8).

Some additional considerations to these definitions should probably be taken into account. For patients who have had no relapse and no marked progression in the MRI for some years before developing a single mild relapse switching to an escalation therapy might not be immediately mandatory. These patients would fulfill the criteria, but it is questionable whether a single mild relapse should be assessed as definite disease progression. These patients should probably be re-evaluated within the next 3–6 months and an escalating therapy should be considered only upon further overt inflammatory disease activity. On the other hand an earlier treatment escalation could be considered for clinically isolated syndrome patients with 9 T2-lesions or one Gd-enhancing lesion developing a relapse within 1 year, even though these patients would not fulfill the criteria of at least one relapse. A relapse within such a short period might be a potential sign of active disease and could be associated with a very poor prognosis.

In the clinical management it should also be considered to test for neutralizing antibodies against IFN beta to become aware of probable treatment failure and to consider spinal MRI in addition to brain MRI for estimating disease severity and prognosis.

In principal, treatment modification should be considered with any deterioration or evidence for new disease activity. Switching from one basic treatment to another does not appear advisable due to the risk of further insufficient benefit although in some individual cases patients might benefit from this strategy. In general, however, the only useful option is a treatment escalation either with fingolimod or natalizumab, the choice of which will depend on the individual circumstances, including contraindications (Table 2) and the patient’s preferences.

**Table 2:** **Contraindications**.

**FINGOLIMOD**
Known immunodeficiency syndrome
Patients with increased risk for opportunistic infections, including immunocompromised patients (including those currently receiving immunosuppressive therapies or those immunocompromised by prior therapies)
Severe active infections, active chronic infections (hepatitis, tuberculosis)
Known active malignancies, except for patients with cutaneous basal cell carcinoma
Severe liver impairment (Child-Pugh class C)
Hypersensitivity to the active substance or to any of the excipients
Some cardiac disorders especially when requiring antiarrhythmic treatment
**NATALIZUMAB**
Hypersensitivity to natalizumab or to any of the excipients
PML, increased risk for opportunistic infections, including immunocompromised patients (including those currently receiving immunosuppressive therapies or those immunocompromised by prior therapies, e.g., mitoxantrone or cyclophosphamide)
Combination with beta-interferons or glatiramer acetate
Known active malignancies, except for patients with cutaneous basal cell carcinoma
Children and adolescents below the age of 18 years

As a next step testing of anti-JCV antibodies is recommended. This test is available free of charge at present. In anti-JCV antibody positive patients the first choice could be fingolimod, but natalizumab could also be considered. However, it should be kept in mind that the risk of developing PML increases after a natalizumab treatment duration of 2 years and especially in patients pre-treated with immunosuppressants. In anti-JCV antibody negative patients fingolimod and natalizumab appear to be similar options, but due to the longer clinical experience with natalizumab, which has been approved since 2006, at the moment this drug could be considered to be the first choice for 2 years of treatment.

Currently there is no evidence about a permanent or residual effect on the immune function of fingolimod in the long-term of discontinuation (for prolonged immune reconstitution: see Johnson et al., 2010). In fact in the majority of patients lymphocyte counts return to basal levels most likely resulting in a complete functional restitution within a period of 3 months after cessation of fingolimod. Although patients’ JCV stages could be taken into account when making decisions about escalation therapy following interferons, there is currently no evidence that the risk of PML would be increased in JCV antibody positive patients receiving fingolimod treatment. In contrast the relation between JCV status and risk has been widely described for natalizumab treatment (Bloomgren et al., 2012).

#### Therapy-naïve patients

**Figure 9:**
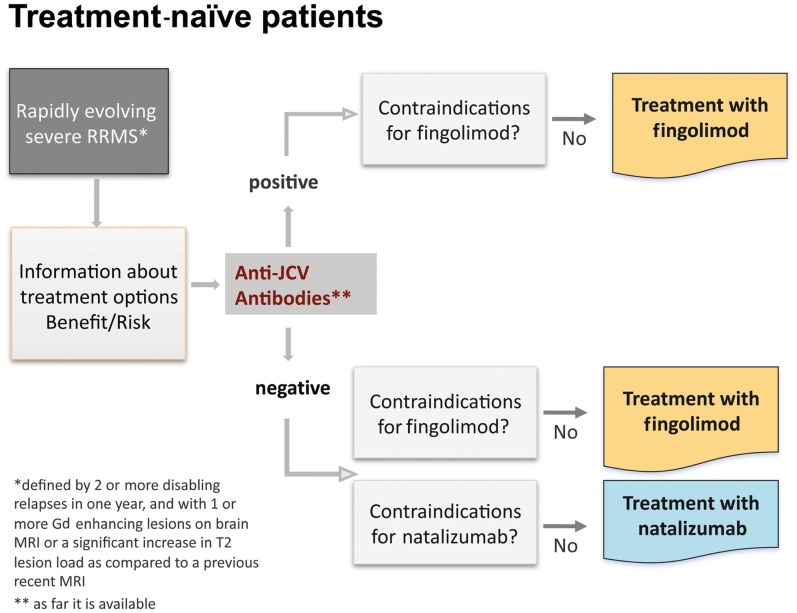
**Algorithm for treatment-naïve patients**.

According to the EU-label, in treatment-naïve patients with rapidly evolving severe RRMS, defined by one or more disabling relapses in 1 year, and with one or more Gd-enhancing lesions on brain MRI, or a significant increase in T2 lesion load as compared to a previous recent MRI, treatment with fingolimod as well as natalizumab is indicated (Figure 9). The first step is to inform the patient about treatment options and consider the benefit/risk-balance. As a second step all contraindications for both treatment options have to be assessed. In the presence of recurrent infections, e.g., herpetic infections, a preventive treatment with acyclovir can be combined with both of these drugs. Mild hepatic impairment is not a contraindication for fingolimod, but requires a careful and frequent (3 monthly) monitoring of liver enzymes (transaminases). The rationale for starting with fingolimod or natalizumab is that only 30% of patients respond to first-line standard treatment and particularly patients with an early and rapidly evolving RRMS may have a bad prognosis if they are not adequately treated from the beginning. Furthermore fingolimod has demonstrated a 25% reduction in ARR vs. IFN beta-1a (*p* = 0.614) in treatment-naïve patients with severe disease at 1 year (Devonshire et al., 2012). The only other alternative would be an unspecific immunosuppressive drug, and this would complicate all other treatment options afterward. A recently completed pharmacoeconomics study showed that even in poor economic countries disability is three times more expensive than the treatment cost (Blahova-Dusankova et al., 2012). This might also be a strong argument for a more expensive, but also more effective therapy from the very onset of highly active disease in this patient group.

#### Switch from natalizumab to fingolimod

**Figure 10:**
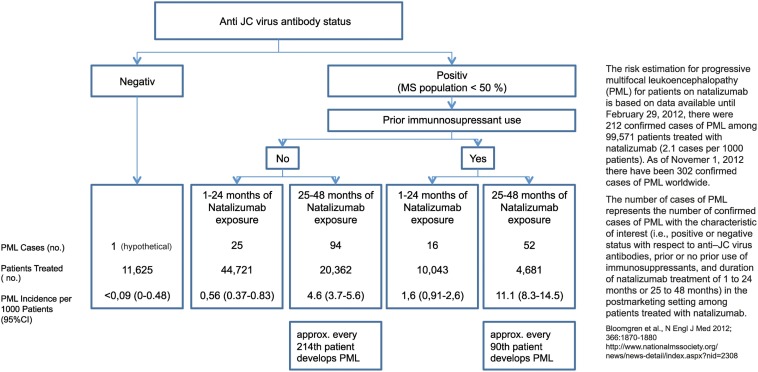
**Risk estimation for progressive multifocal leukoencephalopathy (PML) for patients on natalizumab**.

**Figure 11:**
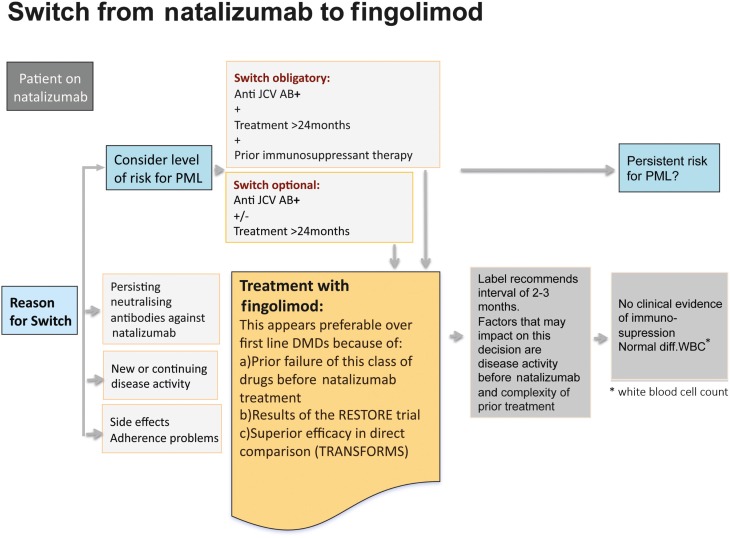
**Reasons that might constitute an indication for switching from natalizumab to fingolimod**.

In a hierarchy of the approved treatments for RRMS fingolimod and natalizumab are equally positioned in Europe. In anti-JCV antibody positive patients with a treatment duration ≥ 2 years and prior immunosuppression a termination of natalizumab treatment is recommended due to the high risk of developing PML (Figure 10). Other reasons that might constitute an indication for switching from natalizumab are persisting neutralizing antibodies against natalizumab, side effects and poor compliance, and also ongoing disease activity (Figure 11). Just one mild symptom alone is not necessarily a reason to terminate natalizumab treatment and supportive evidence from MRI should be used for further treatment decisions.

A switch to fingolimod appears favorable over first-line DMDs for various reasons: as natalizumab is indicated as a second-line treatment, it can be assumed that the patient has received prior basic treatment and is a non-responder to these drugs. Therefore it is unlikely that the patient would like to return to basic treatment and would benefit from it. In line with this are the data from the RESTORE trial (Fox et al., 2011). In this study, all patients were treated for more than 1 year and were relapse-free before treatment with natalizumab was interrupted for 24 weeks. The patients were randomized to continue natalizumab treatment or stop natalizumab and receive either methylprednisolone, intramuscular IFN beta-1a, or subcutaneous GA for up to 28 weeks, followed by re-initiation of natalizumab. MRI scans showed increasing disease activity 12 weeks after stopping natalizumab therapy. Further, 44% of patients on placebo, 53% on GA, 40% on methylprednisolone, and 7% on IFN beta-1a required rescue treatment consisting of high-dose corticosteroids or restarting natalizumab.

Therefore in the vast majority of patients a switch to fingolimod is recommended. First experiences after switching demonstrated that fingolimod is effective in reducing re-occurrence of disease activity after natalizumab discontinuation and causes no severe AEs (Havla et al., 2011).

##### Wash out period

Generally after termination of the treatment with IFN beta or GA no wash out period is necessary, if blood count and liver function are normal. After prior immunosuppressive treatment a wash out period of 6 months is recommended in the summary of product characteristics (SPC).

After natalizumab the label recommends a treatment-free interval of 3 months. As discussed above disease activity begins to return within 3 months after natalizumab treatment interruption (O’Connor et al., 2011). ARRs and Gd+ lesions both increased shortly after interruption and peaked between 4 and 7 months. The extent to which disease activity recurs depends largely on activity before initiation of any disease-modifying treatment together with disease activity before starting natalizumab. It is assumed that a switch from natalizumab to fingolimod is being considered based on clinical need, i.e., to treat patients with otherwise very active disease. Therefore particularly in patients with high disease activity before natalizumab treatment and insufficient treatment response an early switch to fingolimod is recommended. The main goal is to avoid the patient falling into a gap where natalizumab is not active anymore and fingolimod is not yet active. At present we lack specific tests to predict this situation and have to depend on clinical observations. Furthermore, we have to wait for more data informing us whether and to what extent the risk of developing PML is prolonged after termination of natalizumab treatment. A risk of PML with fingolimod itself is unlikely. In clinical trials overall 4000 patients have been treated with fingolimod for a mean duration of 2.5 years, and 140 of them are in the seventh year of treatment. At an average incidence rate of 1.0 PML-case in 1000 patients (based on the natalizumab figures) there should have been at least two PML-cases to date, but so far no cases have been identified under fingolimod treatment without prior, recent natalizumab exposure. Furthermore, at the moment there is no clinical evidence of immunosuppression (all patients had normal differential white blood count).

### Preparatory steps

Before starting fingolimod some preparatory steps have to be taken, which include:

- Detailed information to the patient about mode of action, frequent AEs, contraception, infections, participation in regular checkups, and management plan information to the GP
- Check of disease activity: recent MRI should be available or performed
- History of concomitant diseases and treatments (e.g., antiarrhythmic drugs)
- Neurostatus and physical examination: in the case of relevant findings referral to a specialist (e.g., cardiologist, ophthalmologist); ECG if positive cardiological findings in history or examination
- Lab testing: hematology incl. absolute lymphocyte count (lymphocyte subpopulations); serology incl. liver enzymes, varicella-zoster virus (VZV) titer
- If patient is female: pregnancy test and information of patient about strict and effective contraception
- Depending on individual pre-treatment, appropriate washout period (not applicable for basic immunomodulatory medications).

### Observation period

After evaluation of the preparatory steps fingolimod treatment can be initiated. After an unexplained sudden death of a patient within 24 h after taking the first dose of fingolimod has occurred an extensive re-evaluation of the clinical data on fingolimod has been performed by the US and also the European health agencies (Fazekas, 2012). New recommendations have therefore been made. All patients starting treatment should be observed clinically for a period of at least 6 h for symptoms and signs of bradycardia, AV-block, and hypertension following the initial administration of the drug. This 6 h period can also be used for patient education and explanation of the fingolimod management plan by the MS manager.

Monitoring during the first 6 h after dosing should include (according to the CHMP recommendations)^1^:

- A 12-lead ECG and blood pressure measurement before starting the first dose and after 6 h
- Blood pressure and heart rate measurement every hour after the first dose for 6 h
- During the first 6 h of treatment continuous real time ECG monitoring is recommended.

The patient is discharged after 6 h if the heart rate is unchanged or no longer decreasing. If the patient’s heart rate at the end of the 6 h period is the lowest following first dose administration, the monitoring should be extended by at least 2 h, and until the heart rate increases again.

In those patients with evidence of clinically important cardiological side effects during the first 6 h, monitoring should be extended, including at least overnight monitoring, until resolution.

### Patient management plan

**Figure 12:**
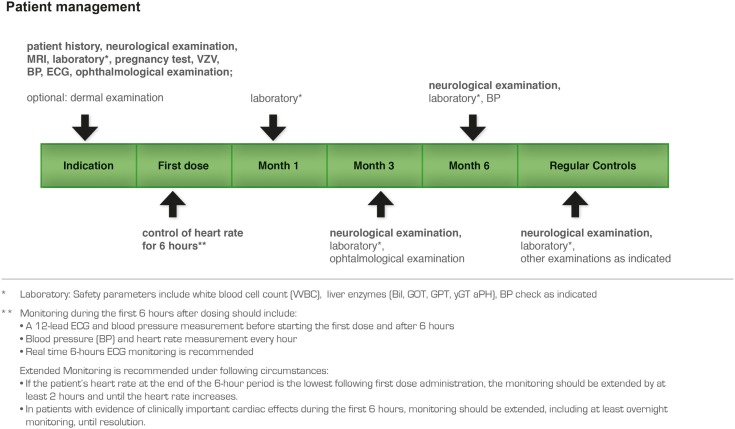
**Recommended patient management plan for treatment with fingolimod**.

According to the risk management plan (Figure 12) the first visit in the long-term follow-up is 1 month after the start of fingolimod treatment and includes laboratory testing (hematology incl. absolute lymphocyte counts, liver enzymes), neurological and physical examination, and history, information to the patient, and assessment of vital signs (blood pressure, heart rate). The second visit after 3 months includes the same examinations and an additional ophthalmological examination to exclude macular edema. The management plan entails subsequent visits every 3 months (laboratory testing, neurological and physical examination, history, and vital signs). Women of childbearing potential must be advised on the potential serious risk for the fetus and the need for effective contraception during treatment with fingolimod, prolonged for about 2 months after treatment termination. If a woman becomes pregnant while on treatment with fingolimod, discontinuation of treatment is recommended. Furthermore, every woman of childbearing potential who receives treatment with fingolimod should be added to the multinational Gilenya^®^ Pregnancy Registry.

Finally, we have to note that the management plan for fingolimod treatment as presented here has been discussed by MS experts exclusively from countries where the indication for fingolimod is “highly active disease.” It was not the aim of the expert meeting and therefore the aim of this paper to apply the introduced criteria also in countries where fingolimod has a first-line indication. Physicians from countries with a first-line indication of fingolimod are therefore recommended to apply criteria that have been developed for their respective regions.

## Future Perspectives

Further long-term experience and the large ongoing and planned study program will deliver more clinical evidence and help us to define the increasingly exact position of the new therapy within our therapeutic armamentarium.

## Conflict of Interest Statement

Ovidiu Bajenaru has participated in meetings sponsored by and received honoraria (lectures, advisory boards, clinical trials) from pharmaceutical companies marketing treatments for MS: Bayer (Schering), Medison, Biogen-Idec, Merck (Serono), Novartis, Sanofi Aventis, TEVA. Thomas Berger has participated in meetings sponsored by and received honoraria (lectures, advisory boards, consultations) from pharmaceutical companies marketing treatments for MS: Allergan, Almirall, AOP, Baxter, Bayer (Schering), Biogen-Idec, Biotest, CSL Behring, Merck (Serono), Novartis, ratiopharm, Sanofi Aventis, TEVA. His institution has received financial support by unrestricted research grants (Allergan, AOP, Biogen-Idec, Berlex, Bayer, Biotest, CSL Behring, Merck Serono, Novartis, ratiopharm, Sanofi Aventis) and for participation in clinical trials in MS sponsored by Bayer Schering, Biogen-Idec, Merck Serono, Novartis, Octapharma, Roche, Sanofi Aventis, TEVA. Fazekas Franz serves on scientific advisory boards for Bayer Schering, Biogen Idec, Genzyme, Merck Serono, Novartis, and TEVA Pharmaceutical Industries Ltd./Sanofi Aventis and has received speaker honoraria from Biogen Idec, Merck Serono, Novartis, and Sanofi-Aventis. Eva Havrdová has received speaker honoraria and payments for consultant services and clinical trials from Biogen Idec, Bayer, Genzyme, GSK, Merck Serono, Novartis, and TEVA. She was supported by Czech Ministry of Education (research program MSM 0021620849 and PRVOUK-P26/LF1/4). Tanja Hojs Fabian declares no conflicts of interest. Alenka Horvat Ledinek declares no conflicts of interest. Gábor Jakab received travel and congress expenses, speaker honoraria from Biogen Idec, TEVA, Serono, Merck, Bayer and Novartis. He served in scientific advisory board for Serono, Merck, TEVA, and Biogen Idec. Tetiana Kobys declares no conflicts of interest. Samuel Komoly has received honoraria for talks and payment for occasional consultancy or research funding from TEVA, Bayer-Schering, Serono, Biogen which manufacture immunomodulatory drugs used in MS. Jörg Kraus received financial support for research activities from Bayer, Biogen Idec, Genzyme, Merck Serono, Sanofi-Aventis, and Novartis. Jörg Krous received personal compensation from Allmiral, Bayer, Biogen Idec, Genzyme, Medtronic, Merck Serono, Sanofi-Aventis, and Novartis for lectures, advisory board participations and consultations. Egon Kurča declares no conflicts of interest. Theodoros Kyriakides declares no conflicts of interest. L'ubomír Lisý declares no conflicts of interest. Ivan Milanov received honoraria in advisory board or lecturer fees from Novartis, Bayer, Pfizer, GSK, UCB, TEVA, Merck, Gedeon Richter, Actavis, CSC Pharmaceuticals. Tetyana Nehrych declares no conflicts of interest. Sergii Moskovko declares no conflicts of interest. Panayiotis Panayiotou declares no conflicts of interest. Saša Šega Jazbec declares no conflicts of interest. Larysa Sokolova declares no conflicts of interest. Radomír Taláb declares no conflicts of interest. Latchezar Traykov received honoraria in advisory board or lecturer fees from Actavis, Gedeon Richter, Novartis, Pfizer, UCB. Peter Turčáni received speaker honoraria, payments for consulting services and clinical trials, and research funding from Bayer, Biogen, Novartis, Merck Serono and TEVA. Norbert Vella has been the recipient of honoraria from Novartis Pharma, financial support to attend meetings from Bial, Biogen Idec, GSK, Merz and Novartis. Nataliya Voloshyna declares no conflicts of interest.

## Footnotes

^1^The full version of recommendations can be read in the SPC.
